# Effectiveness and safety of oral anticoagulant therapy in patients with atrial fibrillation with prior gastrointestinal bleeding: A systematic review and meta-analysis

**DOI:** 10.3389/fcvm.2022.937320

**Published:** 2022-07-27

**Authors:** Jie Zhao, Xiaojuan Wu, Siyuan Li, Qiuping Gu

**Affiliations:** ^1^Department of Cardiovascular Medicine, Wuhan Third Hospital & Tongren Hospital of Wuhan University, Wuhan, China; ^2^Department of Gastroenterology, Ganzhou People’s Hospital, Ganzhou, China; ^3^Department of Hematology, Peking Union Medical College Hospital, Chinese Academy of Medical Sciences, Peking Union Medical College, Beijing, China

**Keywords:** atrial fibrillation, gastrointestinal bleeding, anticoagulation, prognosis, meta-analysis

## Abstract

**Background:**

Gastrointestinal bleeding (GIB) commonly complicates anticoagulant therapy for patients with atrial fibrillation (AF). However, AF patients with prior GIB were excluded from most randomized controlled trials on anticoagulation therapy. Therefore, we conducted a systematic review and meta-analysis to assess the effect of oral anticoagulant (OAC) therapy in this specific population.

**Methods:**

Randomized trials and observational studies reporting the data about the resumption of OAC therapy among AF patients with prior GIB were included. The search was performed in the PubMed and Embase databasesup to March 2022. The adjusted hazard ratios (*HR*s) and 95% confidence intervals (*CI*s) were pooled by a random-effects model with an inverse variance method.

**Results:**

A total of 7 studies involving 57,623 patients were included. Compared with no anticoagulant therapy, OAC therapy was associated with decreased risks of stroke or systemic embolism (*HR* = 0.71, 95% *CI*: 0.59–0.84) and all-cause death (*HR* = 0.66, 95% *CI*: 0.60–0.72), but there was no significant difference in the risk of recurrent GIB (*HR* = 1.22, 95% *CI*: 0.94–1.59). Compared with vitamin K antagonists, non-vitamin K antagonist oral anticoagulants (NOACs) were associated with reduced risks of stroke or systemic embolism (*HR* = 0.61, 95% *CI*: 0.54–0.68), all-cause mortality (*HR* = 0.86, 95% *CI*: 0.75–0.99), major bleeding (*HR* = 0.75, 95% *CI*: 0.66–0.84), and GIB recurrence (*HR* = 0.83, 95% *CI*: 0.72–0.96).

**Conclusions:**

In AF patients with prior GIB, OAC therapy (especially NOACs) demonstrated superior effectiveness compared with no anticoagulant therapy.

## Introduction

It is estimated that there were over 6 million cases of ischemic stroke worldwide in 2013, of which approximately 20% are attributed to atrial fibrillation (AF) (1). Oral anticoagulant (OAC) therapy, including vitamin K antagonists (VKAs) and non-vitamin K antagonist oral anticoagulants (NOACs), has become the backbone of AF management (2–5). However, OAC therapy often comes at the expense of an increased risk of bleeding. Tensions grounded in the history of bleeding events often play a key role in the assessment of all patients taking OAC, particularly in patients with prior gastrointestinal bleeding (GIB).

A large cohort study demonstrated that during the 5-year anticoagulation therapy, 5.7% of elderly patients with AF developed GIB, some of which were at a high risk of mortality (6). Furthermore, although NOACs show a positive role in convenient dosing adjustment and the reduction of risk of stroke, mortality, and intracranial bleeding events, an increased occurrence of GIB has been examined in the same trials (7–11). With such concern, the management of post-GIB medications is extremely difficult in balancing the benefits of stroke prevention against a high perceived risk of recurrent bleeding.

The previous meta-analysis showed that the resumption of OACs in patients following GIB was associated with reduced thromboembolic events and death, with a statistically significant increase in recurrent GI bleeding compared with no-starters (12). However, it was conducted primarily on patients taking VKAs, such as warfarin, and the population was not strictly limited to patients with AF. For patients with AF at a high risk of GIB, a recent network meta-analysis found that resumption of NOACs appeared to be the preferred option compared with warfarin (13). The European Society of Cardiology (ESC) Guidelines recommend that a VKA or another NOAC preparation should be preferred over dabigatran 150 mg two times daily, rivaroxaban 20 mg one time daily, or edoxaban 60 mg one time daily, although lacking strong evidence (14). Therefore, we performed a systematic review and meta-analysis to demonstrate the effectiveness and safety of restarting OAC therapy in AF patients with prior GIB, and further compare the effects of NOACs with VKAs.

## Methods

The meta-analysis was designed and conducted according to the Cochrane Handbook for Systematic Reviews of Interventions (version 6.2) and the Preferred Reporting Items for Systematic Review and Meta-Analysis (PRISMA) 2020 statement. Ethical approval was not required since we only included articles of published data in the public domain.

### Literature search

Two reviewers searched PubMed and Embase database sources from inception to March 2022. The following search terms were used: (1) “atrial fibrillation” OR “atrial flutter,” (2) “gastrointestinal bleeding” OR “gastrointestinal hemorrhage” OR “intestinal bleeding” OR “intestinal hemorrhage” OR “GIB,” and (3) “oral anticoagulant” OR “vitamin K antagonist” OR “VKA” OR “warfarin” OR “non-vitamin K antagonist oral anticoagulant” OR “direct oral anticoagulant” OR “novel oral anticoagulant” OR “NOAC” OR “DOAC” OR “dabigatran” OR “rivaroxaban” OR “apixaban” OR “edoxaban.” The above three categories of search terms were linked by the Boolean command “AND,” and the complete depiction of the search strategy is given in the Supplementary Table 1. In addition, reference lists of the included studies were also searched to identify any studies not found in the initial database search.

### Inclusion and exclusion criteria

The criteria for inclusion were as follows: (1) the study design was a randomized clinical trial (RCT) or an observational (prospective or retrospective cohort) study; (2) the study included AF patients with prior GIB who received VKA or NOACs (dabigatran, rivaroxaban, apixaban, or edoxaban); and (3) effect estimates were adjusted hazard ratios (*HR*s) and 95% confidence intervals (*CI*s), reporting for safety and effectiveness outcomes among patients who resumed OACs and those who did not.

Studies were excluded if the participants had non-GIB [e.g., any bleeding, intracerebral hemorrhage (ICH), major bleeding, and microbleed] or a mixed population without being separately analyzed in the subgroup. In addition, we also excluded certain publication types (e.g., reviews, comments, case reports, case series, letters, editorials, and meeting abstracts) due to insufficient data or study details. If there were overlapping data among two or more studies, we included the one with the largest sample size or the longest follow-up duration. The outcomes considered in our study were SSE, recurrent GIB, and all-cause death. If there are sufficient data on the time to resume anticoagulation, comparisons will be made between different times to resumption. Definitions of the outcomes for each study included in the meta-analysis are shown in Supplementary Table 3.

### Study selection and data abstraction

In this study, two reviewers screened the titles and abstracts of the retrieved studies from the electronic databases. Subsequently, we selected the eligible studies after the full-text screenings based on the pre-defined inclusion criteria. Reviewers compared and discussed results and consulted a third reviewer in case of any disagreement. The reviewer’s abstracted data on the following characteristics: study contexts (first author and year of publication), study design (cohort or RCTs and duration of follow-up), study population (sample size, age, stroke, or bleeding risk prediction scores), outcomes [stroke and systemic embolism (SSE), all-cause death, major bleeding, and recurrent GIB], and measures of association (sample size and the number of events between groups and adjusted *HR*s).

### Study quality assessment

We assessed the quality of the *post hoc* analysis of an RCT or observational cohort by using the Newcastle-Ottawa Scale (NOS) tool. This tool had three domains with a total of nine points: the selection of cohorts (0–4 points), the comparability of cohorts (0–2 points), and the assessment of the outcomes (0–3 points). In this meta-analysis, the NOS of ≥ 6 and < 6 points were scored as moderate-to-high quality and low-quality, respectively (15).

### Statistical analysis

This meta-analysis’s statistical analyses were conducted using the Review Manager version 5.4 software (the Cochrane Collaboration 2014, Nordic Cochrane Center Copenhagen, Denmark).^1^

In this study, significant heterogeneity was indicated by an *I*^2^ value of > 50%, which led to the use of random-effects models and the exploration of a potential source of heterogeneity. When these tests were negative for heterogeneity, fixed-effects models were chosen to calculate pooled *HR*s through the inverse-variance method. In the pooled analysis, the adjusted *HR*s and 95% *CI*s were converted to the natural logarithms (Ln [*HR*]) and their corresponding standard errors (SEs) (Ln [upper CI]-Ln [lower CI]/3.92), which were pooled by a DerSimonian and Laird random-effects model with an inverse variance method. Funnel plots for assessing the potential publication bias for the reported effect estimates were not performed due to the small number of included studies (*n* < 10).

## Results

### Study selection

**FIGURE 1:**
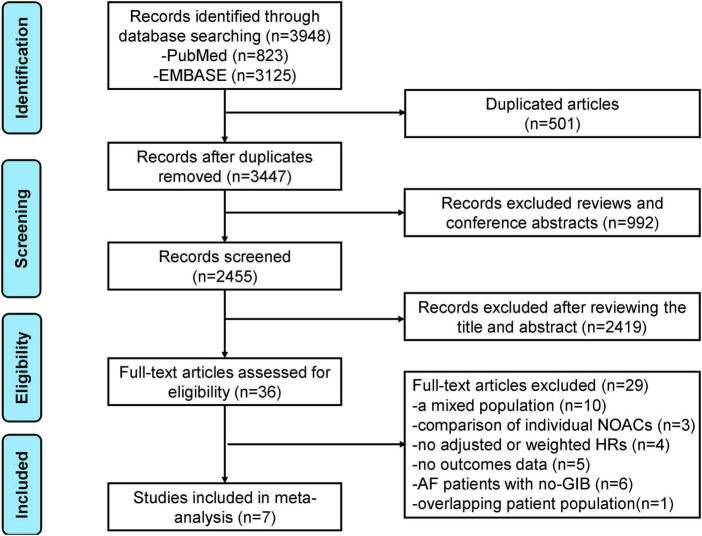
The preferred reporting items for systematic review and meta-analysis (PRISMA) flowchart of this meta-analysis.

The flowchart of literature retrieval is shown in Figure 1. A total of 3,948 records were retrieved from the two databases of PubMed and Embase. After removing duplicates, there were 3,447 bibliographic records identified. Following the elimination of reviews and conference abstracts, the remaining 2,455 articles were under the first phase of the title- and abstract- screenings. After that, 36 remaining studies were potentially available, and further assessed under the full-text screenings. According to the pre-defined inclusion and exclusion criteria, we subsequently excluded 29 studies because (1) studies compared the effects of NOACs (*n* = 3); (2) studies did not report adjusted or weighted *HR*s (*n* = 4); (3) studies focused on a mixed population, and the AF patients with GIB subgroup was not separately analyzed (*n* = 10); (4) studies did not report the studied outcomes (*n* = 5); (5) studies focused on AF patients with no-GIB (*n* = 6); and (6) studies with an overlapping patient population (*n* = 1). Finally, a total of seven studies [one *post hoc* analysis of RCT and six observational cohorts (16-22)] were included in our meta-analysis.

### Baseline characteristics

Baseline characteristics of the included studies are presented in Table 1. Among the included studies, three were from the United States, one was from Denmark, one each from Sweden and Korea, and one from multiple countries. The mean age of patients ranged from 73.5 to 78.3 years, and the sample size was from 784 to 42,048. The study populations in the OAC group across studies were administrated with NOACs (dabigatran, apixaban, rivaroxaban, and edoxaban) or VKAs. A risk of bias evaluation was performed as shown in Supplementary Table 2. All the studies had an NOS of ≥ 6 points suggesting moderate-to-high quality. The inclusion criteria and primary outcomes varied across studies with different adjusted risk factors in the included studies (Supplementary Tables 3, 4).

**TABLE 1:** General characteristics of the included studies in the meta-analysis.

Study-year	Location	Study type	Data source	Study period	Sample size	Mean age (y)	HAS-BLED	CHA2DS2-VASc	Follow-up	NOS
(19)	The United States	Retrospective	anticoagulation clinic of Henry Ford Health System (majority of Southeast Michigan, United States)	January, 2005–December 2010	1,329	75	3 (median)	3 (CHADS_2_, median)	2 years	8
(25)	The United States	Retrospective	IBM MarketScan Research Databases	January 2008–December2017	2,991	77 (warfarin) 78 (NOAC)	NA	NA	6 months	7
(22)	The United States	Retrospective	Truven Health MarketScan Commercial Claims and Encounters Database (Truven Health Analytics, Inc., Ann Arbor, MI, United States)	January 01, 2010–December 31, 2014	1,338	79 (median)	NA	NA	6 months	7
(16)	Korea	Retrospective	Korean Health Insurance Review and Assessment database	January 2010–April 2018	42,048	73 (median)	4	≥4	0.6 year	7
(20)	Sweden	Retrospective	Stockholm Healthcare database	July 2011–June 2018	4,291*^a^*	77.8 (NOAC) 78.4 (warfarin)	2.25 (NOAC) 2.26 (warfarin)	4.21 (NOAC) 4.26 (warfarin)	90 days	6
(17)	America, Europe, Asia Pacific	*Post hoc* analysis of RCT	ARISTOTLE	December 19, 2006–April 02, 2010	784	73.5	NA	NA	1.8 years	8
(19)	Denmark	Retrospective	Nationwide cohort study using Danish registries	January 01, 2005–July 31, 2017	4,842	NA	NA	NA	36 months	7

NA, not available; ICH, intracranial bleeding; GIB, gastrointestinal bleeding; SE, systemic embolism; HIRA, Health Insurance Review and Assessment; CMS, Centers for Medicare & Medicaid Services; RCT, randomized controlled trails;

^a^ Number of patients with a severe GIB after their diagnosis of AF.

### Synthesis of results

#### Effect of OACs vs. no OACs in patients with atrial fibrillation after gastrointestinal bleeding

**FIGURE 2:**
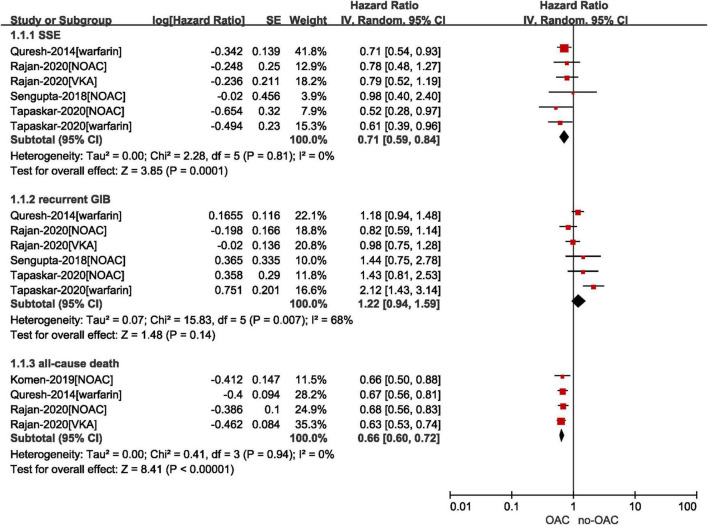
Comparing the effect of OAC with no OAC in AF patients with GIB. AF, atrial fibrillation; GIB, gastrointestinal bleeding; OAC, oral anticoagulants; NOAC, non-vitamin K antagonist oral anticoagulants; CI, confidence interval; IV, inverse of the variance; SE, standard error; SSE, stroke and systemic embolism.

As shown in Figure 2, our pooled results based on the random-effects model showed that compared with no OACs, the use of OACs (NOACs or VKAs) was significantly associated with reduced risks of effectiveness outcomes, including SSE (*HR* = 0.71, 95% *CI*: 0.59–0.84; *I*^2^ = 0%) and all-cause death (*HR* = 0.66, 95% *CI*: 0.60–0.72; *I*^2^ = 0%). There was no significant difference in the risk of recurrent GIB between the two studied groups (*HR* = 1.22, 95% *CI*: 0.94–1.59; *I*^2^ = 68%). We re-analyzed our data, excluding one study at a time to examine if a specific study influenced the results. The risk of recurrent GIB was materially altered by the study conducted by Tapaskar et al. (18).

Specifically, as presented in Supplementary Figures 1, 2, we performed the subgroup analysis by drug regimen (NOACs vs. no NOAC and VKAs vs. no VKAs) on the results of SSE and recurrent GIB. There was no subgroup difference in the risk of recurrent GIB (*p* = 0.56). In terms of the risk of SSE, although no statistically significant subgroup difference was observed (*p* = 0.98), the reduced rate of SSE in patients resuming NOACs was not statistically significant when compared with those who did not take NOACs (*HR* = 0.71, 95% *CI*: 0.50–1.01; *I*^2^ = 0%), whereas the use of VKAs was associated with the decreased risk of SSE compared with no VKAs (*HR* = 0.71, 95% *CI*: 0.58–0.87; *I*^2^ = 0%).

#### Effect of non-vitamin K antagonist oral anticoagulants vs. vitamin K antagonists in patients with atrial fibrillation after gastrointestinal bleeding

**FIGURE 3:**
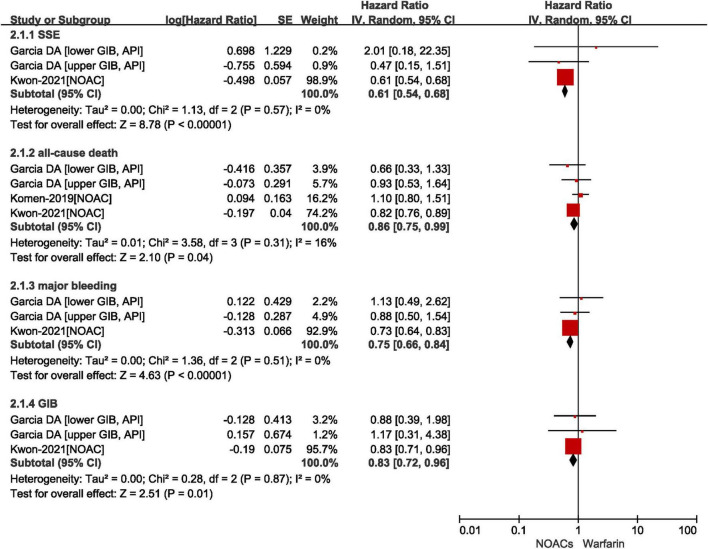
Comparing the effect of NOACs with VKAs in AF patients with GIB. AF, atrial fibrillation; GIB, gastrointestinal bleeding; VKAs, vitamin K anticoagulants; NOAC, non-vitamin K antagonist oral anticoagulants; CI, confidence interval; IV, inverse of the variance; SE, standard error; SSE, stroke and systemic embolism.

A total of three included studies reported the effects of NOACs vs. VKAs in patients with AF after GIB. As shown in Figure 3, our results based on the random-effects model showed that compared with VKAs, the use of NOACs was significantly associated with reduced risks of SSE (*HR* = 0.61, 95% *CI*: 0.54–0.68, *I*^2^ = 0%), all-cause death (*HR* = 0.86, 95% *CI*: 0.75–0.99, *I*^2^ = 16%), major bleeding (*HR* = 0.75, 95% *CI*: 0.66–0.84, *I*^2^ = 0%), and recurrent GIB (*HR* = 0.83, 95% *CI*: 0.72–0.96, *I*^2^ = 0%).

#### Timing of restarting anticoagulation

**FIGURE 4:**
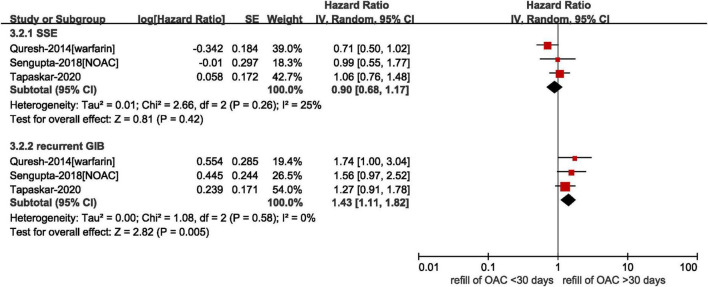
Comparing the effect of different time to resume anticoagulation in AF patients with prior GIB. AF, atrial fibrillation; GIB, gastrointestinal bleeding; OAC, oral anticoagulants; NOAC, non-vitamin K antagonist oral anticoagulants; CI, confidence interval; IV, inverse of the variance; SE, standard error; SSE, stroke and systemic embolism.

Three studies provided data on the timing of resuming anticoagulation. As shown in Figure 4, our results showed that there was no statistically difference in the risk of SSE between refilling anticoagulation within and after 30 days (*HR* = 0.90, 95% *CI*: 0.68–1.17, *I*^2^ = 25%), whereas the resumption of anticoagulation within 30 days was associated with an increased risk of recurrent GIB (*HR* = 1.43, 95% *CI*: 1.11–1.82, *I*^2^ = 0%). It was worth noting that there was an upper-bound limit on the time according to the included studies. Qureshi et al. defined the longest time of discontinuance as 6 months and Tapaskar et al. classified patients based on the first claim within 90 days of discharge (18, 19). In the study conducted by Sengupta et al., the median time to refill a claim for NOACs after GIB was 40 days (22).

## Discussion

In this meta-analysis, we provided evidence that OAC therapy in AF patients with prior GIB was associated with a significant reduction in SSE and all-cause death compared with no anticoagulant therapy, but there was no significant difference in the risk of recurrence GIB between the two groups. In addition, NOACs showed better benefits in SSE, all-cause death, major bleeding, and recurrent GIB compared with VKAs.

When tailoring the treatment of an individual patients with AF, physicians will focus primarily on the risk of stroke and bleeding according to clinical guidelines to ensure patients will derive an immense benefit from anticoagulation. The safety profile of OACs has improved significantly with the widespread use of NOACs, whose favorable risk-benefit profile for stroke, ICH, and mortality was well established, except for GIB (11). Therefore, concern grounded in the rate of GIB often plays a critical role in the prescription. In many cases, AF patients with GIB require temporary discontinuation of OAC therapy in the case of potentially life-threatening bleeding (23, 24). However, coincident with the reduction in bleeding risk, the prevalence of thromboembolism increases. Additionally, many RCTs exclude patients with recent bleeding, making clinical decision-making in specific populations more difficult. Two previous meta-analyses have shown that the resumption of OAC after GIB was associated with a reduced risk of SSE and mortality at the expense of an increased risk of GIB recurrence (12, 25). Recently, Suah et al. (26) performed a subgroup analysis for AF patients with prior GIB and found that NOACs were associated with a reduced risk of ischemic stroke, major bleeding, and GIB compared with warfarin, only using the data by Garcia et al. (17), Kwon et al. (16), and Tapaskar et al. (18). Additionally, a network meta-analysis comparing the effect of resuming NOACs and VKAs in AF patients with prior GIB demonstrated that the resumption of DOACs may be a safer therapy (13).

In terms of effectiveness, our results showed that OACs were associated with a reduced risk of SSE and all-cause death compared with no anticoagulation resumption, which was consistent with two previous meta-analyses. Additionally, subgroup analyses of different drug regimens were performed (NOACs vs. no NOAC and VKAs vs. no VKAs), showing no significant group differences. However, the resumption of VKAs was associated with a reduced risk of SSE compared with non-restarters, but NOACs did not differ. It may be related to the fact that the data of Sengupta et al. (22) were reliant on billing claims, which is likely to underestimate the occurrence of adverse outcomes and patients may not take their medication as prescribed. Additionally, a discrepancy may exist between the date of patients’ resumption and the day when they filed a claim in the insurance plan. Besides, in some situations, physicians may not strictly follow the standard dosing regimen in an attempt to minimize the risk of bleeding, which reduced the effectiveness of NOACs. Further comparisons between NOACs and VKAs demonstrated that NOACs were associated with a better effect on SSE and all-cause death compared with VKAs. However, in the SSE results of Garcia DA et al., there was a wide range of the 95% *CI* due to the small numbers in the lower GIB group. The upper limit of the *CI* for all-cause mortality result was so close to 1 that it needs to be interpreted with caution.

In the risk of bleeding, our results showed no significant difference between patients with the resumption of OAC and those who did not restart in the risk of relapse in GIB, while Tapaskar et al. and Little et al. concluded that the resumption of anticoagulation was associated with a significant increase in recurrent GIB (12, 25). There are several potential reasons why our results are inconsistent with previous findings. First, we restricted our analysis to individuals with AF, while the previous meta-analyses also included patients with venous thromboembolism, ischemic heart disease, and prosthetic valves. A relatively larger number of NOACs-users included in our study should also be considered, as it may make the bleeding risk slightly lower. Additionally, another major part of the difference has been due to the fact that we calculated pooled *HR*s using inverse variance–weighted meta-analysis with random effects. In comparing NOACs with VKAs, Kwon et al. found that NOACs were associated with lower risks of major bleeding than warfarin (16). Similarly, Hu et al. showed that the increased risk of recurrent GIB was associated with the resumption of warfarin but not with NOACs. Our study drew the consistent conclusion that NOACs were associated with a reduced risk of major bleeding and recurrent GIB compared with VKAs. Moreover, for patients with AF, the administration of rivaroxaban was found to be associated with a higher incidence of overall GIB, compared with apixaban or dabigatran (27). Thus, head-to-head comparisons were required to explore whether a similar conclusion would be considered for AF patients with prior GIB.

Finally, we tried to analyze the optimal timing to resume anticoagulation. Due to the small number of relevant articles identified, our results were based on only three articles. In the study by Qureshi et al., compared with patients restarting warfarin after 30 days of interruption, patients who refilled prescriptions within the first week presented a significant higher risk of recurrent GIB (19). Sengupta et al. showed that the resumption of NOACs within 30 days after GIB was not associated with either 90-day or 6-month readmissions for thromboembolic events or recurrent GIB (22). Our results showed that the resumption of anticoagulation within 30 days was associated with an increased risk of recurrent GIB compared with prescription after 30 days. As critical as it is, more high-quality studies are desperately required for making the optimal decision to resume OAC.

## Limitations

Our study still had several limitations. First, we pooled data from observational studies with limited sample size, decreasing our finding’s reliability. Second, the studies did not account for medications, such as non-steroidal anti-inflammatory drugs and aspirin, which might increase the risk of GIB. Similarly, it has been demonstrated that the reduction in GIB associated with NOACs was only statistically significant in patients with no use of proton pump inhibitors and was not significant in those using proton pump inhibitors (27). Third, due to insufficient data, our analysis cannot either derive the specific optimal anticoagulant or elucidate the optimal timing of resumption of the anticoagulant. Fortunately, the “Non-warfarin Oral Anti-Coagulant Resumption After Gastrointestinal Bleeding in Atrial Fibrillation Patients (ClinicalTrials.gov NCT03785080)” is an ongoing RCT investigating how early an NOAC can be safely restarted after acute GIB, which will help provide robust evidence for this issue.

## Conclusion

In AF patients with prior GIB, OAC therapy revealed superior effectiveness profiles compared with no anticoagulant therapy. In addition, NOACs exerted superior effectiveness profiles compared with VKAs.

## Data availability statement

The original contributions presented in this study are included in the article/Supplementary material, further inquiries can be directed to the corresponding authors.

## Author contributions

SL and QG contributed to conception and design of the study. JZ organized the database. XW performed the statistical analysis. JZ wrote the first draft of the manuscript. XW, SL, and QG wrote sections of the manuscript. All authors contributed to manuscript revision, read, and approved the submitted version.

## Footnotes

1
https://community.cochrane.org/
