# Supplementary material for: Effectiveness and safety of oral anticoagulant therapy in patients with atrial fibrillation with prior gastrointestinal bleeding: A systematic review and meta-analysis
Source: Front Cardiovasc Med. 2022 Jul 27;9:937320. doi: 10.3389/fcvm.2022.937320 (PMC9363568; doi:10.3389/fcvm.2022.937320)
Supplement: Supplementary file 1 [file Data_Sheet_1.docx]

**Supplementary Table 1. The search strategies until March 2022**

|  | **Search terms** | **PubMed** | **Embase** |
| --- | --- | --- | --- |
| #1 | atrial fibrillation | 96,776 | 201,455 |
| #2 | gastrointestinal bleeding | 72,967 | 35,911 |
| #3 | gastrointestinal hemorrhage | 59,300 | 75,731 |
| #4 | intestinal bleeding | 28,232 | 4,072 |
| #5 | intestinal hemorrhage | 146,058 | 807 |
| #6 | GIB | 822 | 2,128 |
| #7 | #2 OR #3 OR #4 OR #5 OR #6 | 208,150 | 93,135 |
| #8 | oral anticoagulant | 34,890 | 12,210 |
| #9 | vitamin K antagonist | 9,739 | 5,598 |
| #10 | VKA | 2,295 | 4,985 |
| #11 | warfarin | 32,515 | 105,159 |
| #12 | non-vitamin K antagonist oral anticoagulant | 1,575 | 440 |
| #13 | direct oral anticoagulant | 7,922 | 2,392 |
| #14 | novel oral anticoagulant | 2,776 | 711 |
| #15 | DOAC | 2,230 | 4,586 |
| #16 | NOAC | 1,730 | 3,856 |
| #17 | dabigatran | 6,208 | 19,308 |
| #18 | rivaroxaban | 7,233 | 23,711 |
| #19 | apixaban | 4,758 | 17,234 |
| #20 | edoxaban | 1,944 | 6,681 |
| #21 | #10 OR #11 OR #12 OR #13 OR #14 OR #15 OR #16 OR #17 OR #18 OR #19 OR #20 OR #21 OR #22 | 49,541 | 135,051 |
| #22 | #1 AND #7 AND #21 | 823 | 3,125 |

**Supplementary Table 2. Quality assessment for the included post-hoc analyses and observational studies**

| Included studies | Selection (0-4 points) | | | | Comparability (0-2 points) | | Outcome (0-3 points) | | | Total points* |
| --- | --- | --- | --- | --- | --- | --- | --- | --- | --- | --- |
|  | Representativeness of Exposed Cohort | Selection of Non-Exposed Cohort | Ascertainment of Exposure | Demonstration That Outcome of Interest Was Not Present at Start of Study | Adjust for the important Risk factors | Adjust for other risk factors | Assessment of outcome | Follow-up length | Loss to follow-up rate |  |
| Qureshi et al.-2014 | * | * | * |  | * | * | * | * | * | 8 |
| Sengupta et al.-2018 | * | * | * |  | * | * | * |  | * | 7 |
| Tapaskar et al.-2020 | * | * | * |  | * | * | * |  | * | 7 |
| Kwon et al.-2021 | * | * | * |  | * | * | * |  | * | 7 |
| Komen et al.-2019 |  | * | * |  | * | * | * |  | * | 6 |
| Garcia et al.-2019 | * | * | * |  | * | * | * | * | * | 8 |
| Rajan et al.-2019 | * | * | * |  | * |  | * | * | * | 7 |

＊The Newcastle-Ottawa Scale (NOS) items, with a total score of 9 points, were used to evaluate the quality of the post-hoc analyses of RCTs and observational study which involve the selection of cohorts (0-4 points), the comparability of cohorts (0-2 points), and the assessment of the outcome (0-3 points)

**Supplementary Table 3. Primary outcomes and weighted/****adjusted risk factors in the included studies**

| **Study**  **(First Author-Year)** | **Primary Outcomes** | **Confounders** |
| --- | --- | --- |
| Qureshi et al.-2014 | thromboembolism, recurrent gastrointestinal bleeding, mortality | age, gender, race, Charlson co-morbidity index, number of blood product transfusions, international normalized ratio on admission, and CHADS2 and HAS-BLED scores. |
| Tapaskar et al.-2020 | 180-day hospital re-admissions for recurrent GIB or thrombo-embolic complications | recurrent GIB: demographics, comorbidities, in-hospital management, source of bleeding, and post-discharge anticoagulant resumption; |
| Sengupta et al.-2018 | 90-day hospital readmissions for recurrent GIB and thromboembolic complications | NA |
| Kwon et al.-2021 | ischemic stroke, major bleeding, composite outcome (combined ischemic stroke and major bleeding) events | age, sex, comorbidities, and concomitant medications |
| Komen et al.-2019 | All-cause mortality | age, sex, the individual components of the Charlson Comorbidity Index, the CHA2DS2-VASc score, the modified HAS-BLED score, baseline medication as described above, the year of inclusion |
| Garcia et al.-2019 | ischemic or hemorrhagic stroke or systemic embolism | **Stroke outcomes:** weight, diabetes, hypertension, moderate valvular disease, prior stroke/transient ischemic attack (TIA)/systemic embolism, type of AF, and prior vitamin K antagonist (VKA) use.  **Death outcomes:** sex, weight, systolic blood pressure, diastolic blood pressure, hypertension, moderate valvular disease, left bundle branch block, prior MI, prior stroke/TIA/systemic embolism, anemia, smoking, prior VKA use, New York Heart Association (NYHA) class, CHADS2 score, and renal function.  **Bleeding outcomes:** sex, coronary artery disease (CAD), prior MI, prior non-GI bleeding, anemia, CHADS2 score, and renal function. Adjustment variables for MI included diabetes, CAD, prior MI, NYHA class, and renal function. |
| Rajan et al.-2022 | all-cause mortality, stroke/thromboembolism, recurrent gastrointestinal bleeding | NA |

**Supplementary Table 4. The inclusion criteria of studies**

| **Study**  **(First Author-Year)** | **Inclusion criteria** |
| --- | --- |
| Qureshi et al.-2014 | (1) Patients who had commenced using warfarin for at least 1 year and with at least 2 prescriptions of warfarin within 3 months of contact with a physician were enrolled. (2) The database query was further narrowed using ICD-9 code 427.3, 427.31, and 427.32 for AF. |
| Tapaskar et al.-2020 | (1) Adults aged 18 years and older with at least 1 inpatient or 2 outpatient claims for AF occurring on separate dates within 12 months before the index prescription ﬁll date for either dabigatran, rivaroxaban, apixaban, or warfarin;  (2) Patients were enrolled continuously in the insurance plan for at least 12 months before the index prescription ﬁll date through 180 days after the index hospitalization discharge;  (3) Patients were hospitalized with a primary discharge diagnosis of GIB after the index prescription ﬁll date for 1 of the anticoagulants;  (4) 1 or more prescriptions for an anticoagulant was ﬁlled within 3 months before the hospitalization for GIB;  (5) Patients were discharged alive from the index hospitalization. |
| Sengupta et al.-2018 | (1) Adult patients older than 18 years of age with at least 1 outpatient or inpatient claim for AF ([ICD-9] Clinical Modiﬁcation codes 427.3, 427.31, and 427.32) occurring within 12 months before an index prescription ﬁll date for either dabigatran, rivaroxaban, or apixaban.  (2) Patients subsequently hospitalized for GIB within 365 days of their index DOAC prescription ﬁll date and within 1 month of the end of a prescription claim for their DOAC.  (3) patients with continuous enrollment for 12 months before their initial prescription claim and 6 months after hospitalization for GIB were included. |
| Kwon et al.-2021 | (1) AF patients with OAC prescriptions between January 2010 and April 2018.  (2) Patients who had a history of GIB before the index treatment of OACs. |
| Komen et al.-2019 | All patients had a prior diagnosis for AF (I48) (ICD-10 codes) with a severe GIB. |
| Garcia et al.-2019 | (1) Patients in the ARISTOTLE trail with a history of GIB.  (2) GIB was considered to be recent if it had occurred less than 1 year prior to randomization. |
| Rajan et al.-2022 | All patients diagnosed with AF and receiving oral anticoagulants prior to admission to hospital with gastrointestinal bleeding were included |


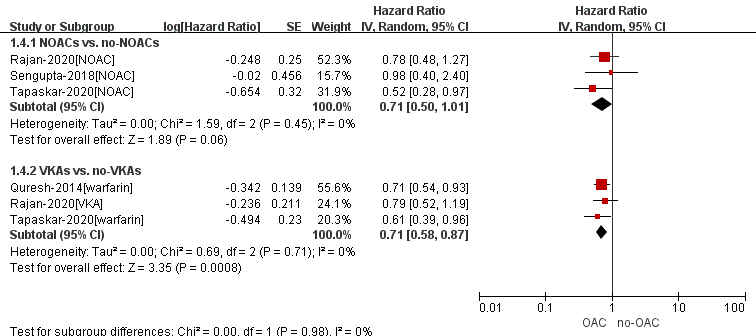


**Supplemental Figure 1: Subgroup analysis by drug regimen (NOAC, VKAs) on the risk of SSE.**

GIB, gastrointestinal bleeding; NOACs, non-vitamin K antagonist oral anticoagulants; VKAs, vitamin K anticoagulants; CI, confidence interval; IV, inverse of the variance; SE, standard error; SSE, stroke and systemic embolism


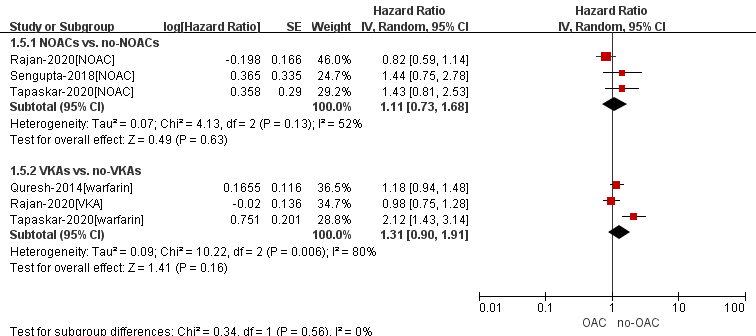


**Supplemental Figure 2: Subgroup analysis by drug regimen (NOAC, VKAs) on the risk of recurrent GIB.**

GIB, gastrointestinal bleeding; NOACs, non-vitamin K antagonist oral anticoagulants; VKAs, vitamin K anticoagulants; CI, confidence interval; IV, inverse of the variance; SE, standard error; SSE, stroke and systemic embolism;
